# Use of wearables to measure the effects of long COVID on activities of daily living and their relationship to perceived exertion, occupational performance, and quality of life

**DOI:** 10.3389/fpubh.2025.1519204

**Published:** 2025-02-19

**Authors:** Lucía Hernández-Hernández, Paula Obeso-Benítez, Sergio Serrada-Tejeda, Patricia Sánchez-Herrera-Baeza, Ma Pilar Rodríguez-Pérez, Marta Pérez-de-Heredia-Torres, Rosa María Martínez-Piédrola, Jorge Martín-Hernández

**Affiliations:** ^1^Department of Physical Therapy, Occupational Therapy, Physical Medicine and Rehabilitation, Research Group in Evaluation and Assessment of Capacity, Functionality and Disability (TO+IDI), Universidad Rey Juan Carlos, Alcorcón, Spain; ^2^Fisioterapia Amaia Rodríguez, Gipuzkoa, Spain

**Keywords:** long COVID, activities of daily living, perceived exertion, occupational performance, quality of life, wearables, occupational therapy

## Abstract

**Introduction:**

This study introduces a novel approach to understanding the impact of long COVID symptoms on daily life by integrating wearable devices to assess their influence on physical and mental quality of life, as well as perceived performance and satisfaction in daily activities.

**Methods:**

By leveraging technology such as accelerometers and pulse oximeters alongside assessment tools like the SF-12 Health Survey, the Canadian Occupational Performance Measure, and the Borg Scale, this research provides a comprehensive analysis that advances the field of occupational therapy.

**Results:**

An analytical observational study with 10 participants with long COVID and 10 healthy controls revealed that individuals with long COVID took significantly longer to complete tasks such as setting the table, sweeping, and climbing stairs, compared to the control group. Participants with long COVID also reported higher perceived exertion during all activities, as well as significantly worse physical health-related quality of life and lower satisfaction and performance in daily activities. Notably, perceived exertion correlated with reduced physical quality of life and diminished satisfaction and accomplishment in occupational tasks.

**Discussion:**

These findings emphasize the critical need for occupational therapy interventions to reduce perceived exertion, which could improve physical quality of life and enhance performance and satisfaction in daily activities for individuals with long COVID.

## Introduction

The National Institute for Health and Care Excellence defines long COVID (LC) as a complex, multi-organ condition affecting individuals who have experienced COVID-19 and continue to exhibit symptoms beyond the acute phase of the disease. This includes ongoing symptomatic COVID-19 (4 to 12 weeks) and post-COVID-19 syndrome (12 weeks or more) (1, 2). The prevalence is estimated to range between 10 and 20% of infected individuals (3–5). Key risk factors for developing LC included age, female sex, obesity, and the severity of the initial infection (6, 7).

The most characteristic symptoms of LC include dyspnea, fatigue, post-exertional malaise, cough, chest pain, sleep disorders, headache, psychological problems, such as anxiety and depression, and cognitive dysfunctions, such as difficulties with concentration and memory (3, 8, 9). Rahmati et al. reported that 2 years after recovery from SARS-CoV-2 infection, 41.7% of survivors continue to suffer from neurological, physical, and psychological sequelae (10). Specifically, among the persistent symptoms, cardiorespiratory issues are particularly disabling, ranging from pulmonary fibrosis to vascular conditions of varying severity. These conditions are hypothesized to result from virus-mediated or autoimmune dysfunction of intrathoracic chemoreceptors and mechanoreceptors or brainstem involvement (8). Such abnormalities can manifest as dyspnea, ranging from mild to severe forms, with permanent oxygen desaturations requiring supplemental oxygen, often accompanied by generalized weakness (11). Additionally, residual viral fragments can trigger a cytokine storm, leading to an inflammatory overactivation inadequate for combating the disease (12). Post-exercise fatigue also contributes to pronounced exercise intolerance, exacerbating symptoms hours or even days after physical activity (13). The variability in duration, severity, and fluctuation of these symptoms significantly impacts quality of life, functional status, and mood (14).

Despite the growing recognition of LC, limited studies have investigated its impact on activities of daily living (ADLs) (15–17). Available research suggests that fatigue is one of the primary symptoms influencing ADLs (16–19). LC-related participation restrictions affect both basic and instrumental ADLs, leading to dysfunctions in occupational performance and roles (20, 21). According to a 2020 national survey conducted by the Spanish Society of General Medicine, 50% of individuals with LC reported significant disability. Among the most affected ADLs were instrumental tasks such as leisure and domestic activities, work-related functions, and basic ADLs like personal hygiene (22). Pizarro-Pennarolli et al. identified nine studies indicating a decline in ADL performance post-SARS-CoV-2 infection, assessed using tools such as the Barthel Index, the Activities of Daily Living Score, and the Functional Independence Measure (23).

Several scales have been proposed to evaluate the functional limitations caused by LC, including the COVID-19 Yorkshire Rehabilitation Scale (24) and the Post-COVID-19 Functional Status Scale (25). Although these tools generally provide insights into patients’ conditions, there remains a need for more objective analyses of LC’s impact on functionality (25, 26). Additionally, scales designed to address patient-reported symptoms, such as fatigue, are essential (27). Notably, the Borg Scale has been widely used to measure subjective respiratory exertion in LC populations (28, 29).

In occupational therapy for LC, tools like the Canadian Occupational Performance Measure (COPM) are used to assess the impact of the condition on daily life (30, 31). Findings show that work-related occupations are impaired in 97% of cases, while leisure activities are affected in 93% (20). Analyzing the components of performance affected by LC is crucial for linking them to occupational dysfunctions and designing targeted interventions to improve outcomes. Evaluating both cardiorespiratory parameters and the subjective perception of physical exertion is crucial for understanding their influence on quality of life, as well as changes in perceived performance and satisfaction with daily occupations.

Wearable devices have recently emerged as promising tools for their simplicity, portability, and ability to provide real-time monitoring. These devices could offer significant benefits for tracking LC (32). Accelerometers, for instance, have been widely used in various populations and conditions to measure physical activity levels and have shown associations between LC symptoms and physical activity in recent studies (33). However, no studies to date have explored the relationship between physical activity and the performance of specific ADLs in LC patients, highlighting a significant gap in the current research.

This study aims to address this gap by analyzing the impact of LC symptoms on ADLs using wearable devices and assess their relationship with physical and mental quality of life, as well as perceived changes in performance and satisfaction in daily occupations.

## Materials and methods

### Design

An analytical observational case–control study was carried out, following the recommendations established by the STROBE statement (34).

The study was approved in May 2022 by the ethical committee of the Universidad Rey Juan Carlos, with internal reference ENM21/213003202210122, in accordance with the ethical principles for medical research involving human subjects of the Declaration of Helsinki adopted at the 18th Assembly of the World Medical Association (WMA) (Helsinki, Finland, June 1964) and its subsequent revisions (35). Participants were informed of the study process and were given the informed consent form to accept and sign if they were interested in being included in the research.

### Sample

The sample of the experimental group (EG) was obtained by non-probabilistic consecutive sampling, through the registry provided by the Spanish Society of General Family Physicians (Sociedad Española de Médicos Generales de Familia); while that of the control group (CG) was obtained by convenience and matched for age and sex. The inclusion criteria for the EG were: (1) persons aged between 18 and 65 years, (2) diagnosed with acute-phase COVID-19 disease via Polymerase Chain Reaction (PCR) and/or positive serology and with LC determined by medical diagnosis, (3) resident in Spain, and (4) accepting and signing the informed consent form. The CG included (1) persons aged between 18 and 65 years, (2) who had not had COVID-19 or, if they had had it, did not present LC, (3) were resident in Spain, and (4) accepted and signed the informed consent form.

Those individuals in both groups who presented cognitive, physical, or sensory deficits that prevented them from understanding the questionnaires or carrying out the activities were excluded.

### Measures

This section details the tools and instruments selected for the assessment of quality of life, perceived changes in performance and satisfaction in daily occupations, physical exertion, intensity of physical activity, and cardiorespiratory parameters. Each measure was chosen based on its established validity, reliability, and applicability to provide a comprehensive analysis of the impact of long COVID on daily living.

The SF-12 Health Survey was used to evaluate quality of life. This instrument is a concise, 12-item version of the widely recognized SF-36 Health Questionnaire. The SF-12 items use Likert-type response scales with 3 to 5 options (36). Studies have confirmed its validity and reliability, reporting internal consistency coefficients exceeding 0.70 and significant correlations with the SF-36, making it a robust tool for measuring physical and mental health (37). In addition, other studies have used this instrument with people with LC (36, 38, 39).

The Canadian Occupational Performance Measure (COPM) was utilized to measure perceived changes in performance and satisfaction across daily occupations. This client-centered interview tool assesses self-care, productivity, and leisure through a structured five-step process. Participants identify key occupations they wish to address and rate their performance and satisfaction on a 10-point scale (30). COPM has demonstrated strong validity and reliability, as well as high sensitivity to changes over time, making it particularly suited for identifying occupational challenges and tracking rehabilitation outcomes (40). Different authors have administered this assessment tool in populations with LC (41, 42).

The Borg Scale was employed to measure the subjective perception of physical exertion. This visual analog scale ranges from 0 to 10, where 0 represents “rest” and 10 indicates “maximal exertion.” It is widely used in exercise physiology and rehabilitation studies to quantify perceived effort during physical activity (43). This scale has also been previously used with the study population (13, 44).

The ActiGraph wGT3X-BT, manufactured by ActiGraph (Pensacola, Florida, USA), is a triaxial accelerometer designed to estimate the intensity and duration of physical activity. This device measures acceleration across three orthogonal axes (x, y, z), capturing three-dimensional movements with high precision. Programmed at a sampling frequency of 30 Hz, it records data in predefined intervals, enabling the classification of activity into intensity levels, including light, moderate, vigorous, and very vigorous. Additionally, it provides estimates of energy expenditure expressed in metabolic equivalents (METs) and calculates the total time spent performing the activity, as well as the time spent at each intensity level. The accelerometer operates by detecting acceleration forces generated by body movements, which are converted into electrical signals by piezoelectric sensors. These signals are then processed and analyzed to identify movement patterns and quantify physical activity. The device has demonstrated high validity and reliability across various populations, including those with chronic conditions (45, 46). For data processing, ActiLife 6 software, also developed by ActiGraph (Pensacola, Florida, USA), was used, enaibling detailed analyses of activity patterns and trends. The ActiGraph has been used in other studies with people with LC (47, 48).

The Handheld SP-20 Pulse oximeter measures arterial oxygen saturation (SpO2) and heart rate (HR) at approximately 2-s intervals. Its principle of operation is based on spectrophotometry, utilizing beams of red and infrared light emitted by a diode through tissues with adequate blood perfusion, such as the fingertip or earlobe. The light absorbed by oxygenated and deoxygenated hemoglobin is detected by a photodiode, allowing the calculation of the percentage of oxygen bound to hemoglobin in arterial blood. The SP-20 is compact and user-friendly, making it suitable for research. In addition to its accuracy in measuring cardiorespiratory parameters, it provides real-time data, which is crucial for monitoring physiological responses during physical activities and at rest (49). The pulse oximeter is an important instrument for people with LC, and different authors have used it in their studies (49, 50).

### Procedure

Prior to beginning the study, the researchers selected the ADLs to be observed. For this purpose, the complete list of all the available ADLs was carefully analyzed, considering the classification of activities of the Assessment Motor and Process Skills (AMPS) as a starting reference (51). The four selected ADLs were (1) Setting the table for four people, (2) Sweeping, (3) Putting on shoes and socks and (4) Climbing stairs (22 steps). The criteria used for their selection were the number of associated aspects and functional requirements, their feasibility due to the characteristics of the environment, their level of difficulty and physical exertion, the degree of efficiency and safety, and the need for assistance for each of them. Moreover, the activities were selected to avoid any gender bias.

**Figure 1:**
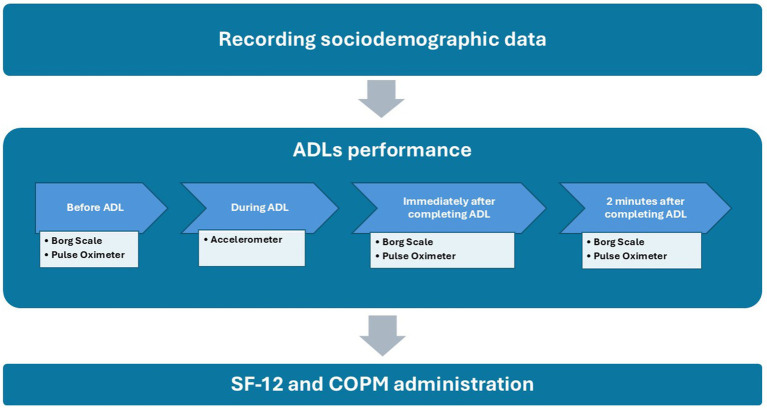
Fieldwork diagram. ADLs, activities of daily living; SF-12, SF-12 Health Survey; COPM, Canadian Occupational Performance Measure.

The patients were referred through the Spanish Society of General Family Physicians and were contacted by e-mail, informing them about the investigation. Each participant was assigned an anonymized identification code. This number was recorded in a register with the aim of identifying them in the successive assessments and was kept during the course of the study, to be used during data collection and analysis. Once each participant had provided consent, a face-to-face appointment was scheduled for data collection. Thus, sociodemographic data were recorded, such as age, sex, height and weight. Subsequently, the four ADLs were performed, always in the same order, in a controlled space that simulated a home environment. While executing these activities, the subject wore an accelerometer which was located over the hip (the accelerometer was programmed and placed prior to beginning the ADLs). The Borg Scale data were collected at rest, prior to beginning the activity, together with HR and SpO2 using the pulse oximeter placed on the index finger. These data were collected again immediately after completing the activity and 2 min after its completion. Once the ADLs were completed, the SF-12 and COPM questionnaires were administered through an interview with the participant. The fieldwork diagram can be seen in Figure 1.

Data collection took place between April and May 2022 in the laboratories of the Faculty of Health Sciences of the Rey Juan Carlos University, Alcorcón (Madrid), and in the Amaia Rodríguez Physiotherapy Center, Zumárraga (Guipúzcoa), both in Spain. The patients were always scheduled in the same time slot and, as they were conducted in two different geographical settings, the characteristics of the environment were homogenized, replicating the measurements, distances, materials, objects and distracters. Each of the activities had a comprehensive protocol describing the objects used and their measurements, the location of each object and the participant’s starting position, the instructions given to the participant and the actions and sequences that should occur in each activity. The complete protocol can be found in the Supplementary material.

### Data analysis

The statistical analysis of the variables was conducted using IBM SPSS Statistics for Windows, version 27.0 (Copyright© 2013 IBM SPSS Corp.). To describe the sample characteristics and study variables, descriptive statistics were applied. Quantitative variables were expressed as means and standard deviations (SD), complemented with 95% confidence intervals (CI) to provide an estimation of the population parameters. Qualitative variables were reported as frequencies and percentages, allowing for a clear depiction of categorical data distribution.

The analysis of normality was conducted to determine whether the data followed a normal distribution, a critical step in selecting the appropriate statistical tests for hypothesis testing. This analysis is essential because parametric tests assume normality in the data distribution to provide valid results. The Shapiro–Wilk test was used to assess normality due to its sensitivity, especially with small sample sizes, as it compares the sample distribution to a perfectly normal distribution (52).

Depending on the results of the normality test, appropriate statistical tests were employed for comparisons. For normally distributed independent samples, the Student’s t-test was applied, as it is widely recognized for its sensitivity to detect mean differences between two groups (53). For non-normally distributed variables, the Mann–Whitney U test was utilized, a nonparametric alternative that does not rely on distributional assumptions (53). To evaluate correlations between variables, the Spearman rank correlation coefficient was used, suitable for assessing monotonic relationships when at least one variable does not meet parametric assumptions (54).

Effect sizes were calculated to quantify the magnitude of observed differences, following best practices for reporting statistical findings. For parametric variables, Cohen’s d was used, with thresholds of 0.2, 0.5, and 0.8 representing small, medium, and large effect sizes, respectively (55). For nonparametric variables, Rosenthal’s r was employed, with similar interpretative thresholds (56). These effect size measures provide an intuitive understanding of the practical significance of the results beyond mere statistical significance.

Statistical significance was set at *p* < 0.05, with a confidence level of 95%, in line with conventional standards for hypothesis testing in biomedical research (57).

## Results

**Figure 2:**
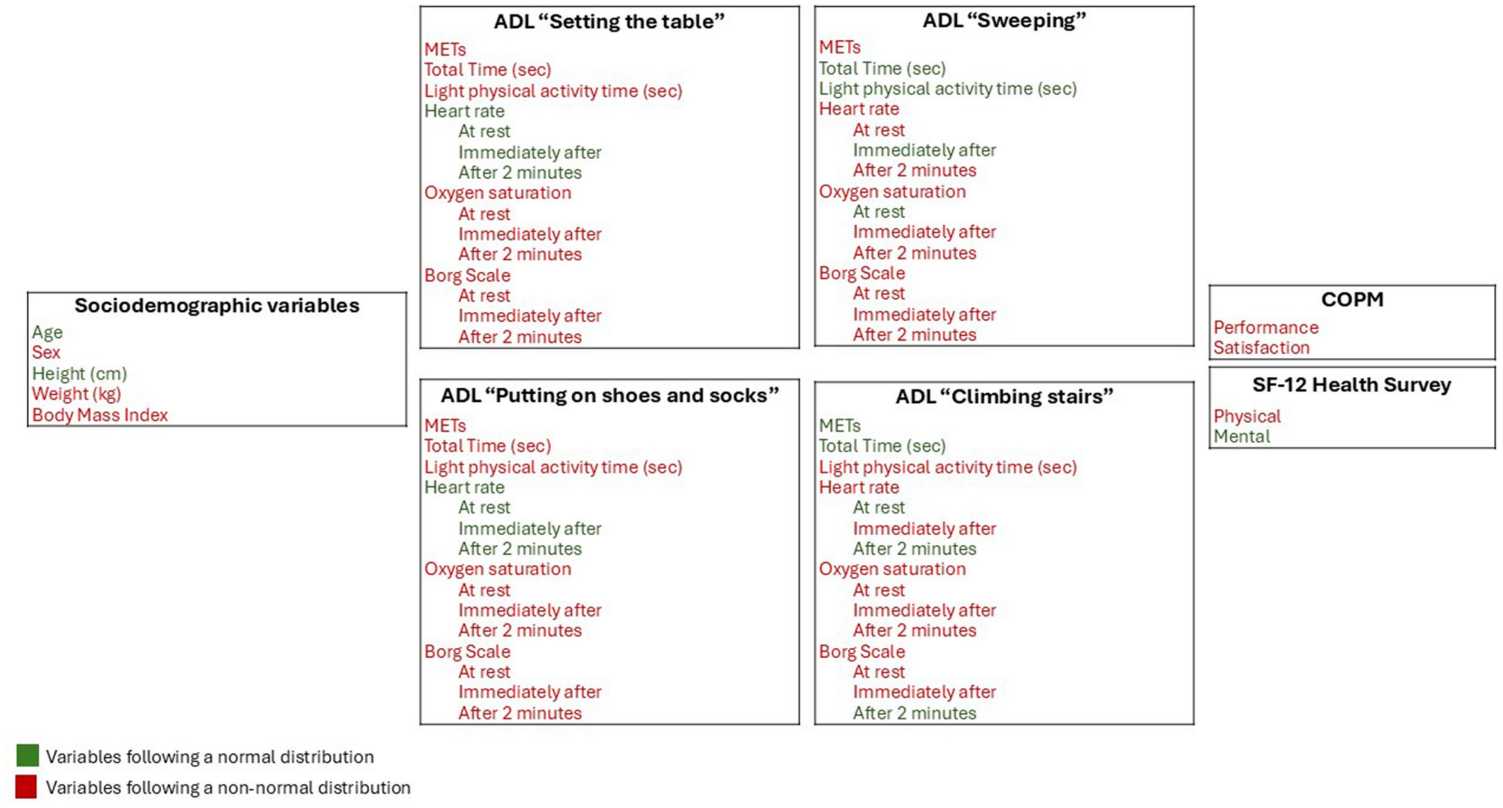
Normality analysis of the study variables. ADL, activity of daily living; METs, metabolic rate measurement unit.

After applying the necessary statistical methods to analyze the normality distribution of the study variables, it was determined which of them follow a normal distribution and which do not. The results of this analysis are presented in Figure 2.

The total sample consisted of 20 participants: 10 in the control group without LC and 10 in the experimental group diagnosed with LC. The sociodemographic data of the sample are presented by groups in Table 1. The analyses indicate that there were no significant differences in the collected variables, suggesting that the sample was homogeneous. This means that the possible differences between the groups in the variables studied in this work are not due to differences in the studied sociodemographic variables.

**Table 1:** Sociodemographic data of the sample by groups (*n* = 20).

	CG (*n* = 10)	EG (*n* = 10)	*p* value
**Age**	47.70 ± 16.14	47.40 ± 7.71	0.959
**Sex**			
Male (%)	3 (30)	2 (20)	NA
Female (%)	7 (70)	8 (80)	NA
**Height (cm)**	165.90 ± 10.23	165.10 ± 8.12	0.849
**Weight (kg)**	64.90 ± 8.74	75.30 ± 16.50	0.088
**BMI**	23.64 ± 2.90	27.47 ± 4.55	0.038*
Normal weight (%)	7 (70)	2 (20)	NA
Overweight (%)	3 (30)	5 (50)	NA
Obesity (%)	0	3 (30)	NA

CG, Control group; EG, Experimental group; BMI, Body mass index; NA, Not applicable*p < 0.05.

Applying the instruments described in the ‘Measures and Procedure’ section, the following variables were obtained: energy expenditure (METs), total time dedicated to performing ADLs, time spent in light physical activity, heart rate, oxygen saturation, and perceived exertion in the selected activities. Additionally, perception of occupational performance and quality of life were included. The differences between both groups were analyzed, and the results of this analysis are presented in Supplementary Table 1.

The results reveal statistically significant differences in the total time taken to complete the activities of laying the table, sweeping, and climbing stairs, with individuals with LC spending more time on all three activities. The effect size of the difference was large in the sweeping and stair climbing activities (d ≥ 0.8) and moderate in the laying the table activity (d ≥ 0.5). In addition, in the activities of laying the table and sweeping there were also significant differences in the time spent doing light physical activity, with more time spent by people with LC doing light intensity activities compared to people without LC. The effect size was large for the sweeping activity (d ≥ 0.8) and moderate (r ≥ 0.5) for laying the table.

In terms of perceived exertion, significant differences are observed in all four ADLs. The effect size difference was generally moderate (r ≥ 0.5). In all the ADLs and measurement times, people with LC experienced a greater perception of exertion compared to the control group. People with LC presented worse perception and satisfaction with their occupational performance than the control group. Significant differences were found in the two variables (*p* < 0.05) with a large effect size for both (r ≥ 0.8).

Regarding quality of life, differences were found in the physical dimension with a large effect size (r ≥ 0.8), with people with LC having a worse quality of life related to physical health. Table 2 and Supplementary Tables 2, 3 show the analysis of correlations between perceived exertion, quality of life, occupational performance, heart rate and oxygen saturation.

**Table 2:** Correlation between perceived exertion in four activities of daily living, quality of life and occupational performance, for each group.

		EG (*n* = 10)				CG (*n* = 10)			
Borg scale		SF-12		COPM		SF-12		COPM	
		Physical	Mental	Performance	Satisfaction	Physical	Mental	Performance	Satisfaction
ADL_Table
At rest	−0.324	0.293	−0.550*	−0.157	−0.406	0.290	0.292	0.059
Immediately after	−0.612*	0.373	−0.589*	−0.105	0.165	−0.220	0.194	−0.502
After 2 min	−0.477*	0.330	−0.589*	−0.077	−0.406	0.290	0.292	0.059
ADL_Sweeping									
At rest	−0.606*	0.355	−0.635*	−0.194	−0.406	0.290	0.292	0.059
Immediately after	−0.868*	0.285	−0.756*	−0.583*	0.069	−0.208	0.133	−0.236
After 2 min	−0.440	0.361	−0.549*	−0.178	−0.406	0.290	0.292	0.059
ADL_Shoes									
At rest	−0.507*	0.298	−0.680*	−0.280	−0.406	0.290	0.292	0.059
Immediately after	−0.492*	−0.025	−0.630*	−0.529*	0.180	−0.257	0.119	−0.372
After 2 min	−0.593*	0.183	−0.755*	−0.326	−0.406	0.290	0.292	0.059
ADL_Stairs									
At rest	−0.451*	0.198	−0.625*	−0.236	−0.046	0.290	0.292	0.059
Immediately after	−0.685*	0.269	−0.727*	−0.434	0.189	−0.352	0.007	−0.382
After 2 min	−0.367	0.177	−0.653*	−0.286	−0.046	0.290	0.292	0.059

*p < 0.05; ADL_Table, “Setting the table”; ADL_Sweeping, “Sweeping”; ADL_Shoes, “Putting on your shoes”; ADL_Stairs, “Climb stairs”; COPM, Canadian Occupational Performance Measure; SF-12, SF-12 Health Survey.

After analysis of the correlation in the experimental group, it was found that the correlation between perceived exertion and the physical dimension of quality of life was negative; therefore, greater perceived exertion during an activity is related to a poorer quality of life in terms of physical health. A negative correlation was also found between perceived exertion and occupational performance, i.e., the lower the perceived exertion during an activity, the better the occupational performance. Conversely, perceived exertion was not correlated with heart rate or oxygen saturation in any of the four activities. A higher or lower heart rate, as well as a higher or lower oxygen saturation, was not correlated with a higher or lower perception of exertion in this study population. These results are shown in Supplementary Tables 2, 3.

## Discussion

The present research represents a significant advance in understanding the impact of LC on ADLs by analyzing cardiorespiratory and physical activity parameters using wearable devices in comparison to individuals without LC. Specifically, the findings show that patients with LC require significantly longer times to perform tasks such as laying the table (149.60 ± 47.42), sweeping (206.30 ± 54.60), and climbing stairs (19.40 ± 4.25) compared to the control group. This result aligns with Wright et al. (58) who proposed that increased physical activity may exacerbate LC symptoms. However, physical exercise has documented positive effects, including improved immunological function through increases in neutrophils, monocytes, T lymphocytes and macrophages; higher levels of immunoglobulins (IgA, IgM and IgG), crucial for lung infections; and regulation of C-reactive proteins levels, which are elevated in LC patients. This underscores the need for progressively controlled exercise prescribed by qualified professionals to mitigate these challenges (59). These findings suggest that individuals with LC may allocate additional time to ADLs as a compensatory strategy to reduce discomfort and perceived exertion.

A significant finding of this study is the elevated perception of exertion in individuals with LC, noted at rest, immediately after, and 2 min post-ADL completion. This heightened perception likely stems from the chronic fatigue and post-exertional discomfort characteristic of this population, as reported by Twomey et al. (27). Recent studies show that increased feelings of fatigue can make it difficult to perform ADLs (60), and to improve the management of these symptoms it is essential that occupational therapy interventions emphasizing energy conservation strategies, environmental adaptations, and activity simplifications, such as dividing tasks into smaller steps and scheduling adequate rest periods (26, 61). Implementing these strategies could enable LC patients to complete ADLs in less time, with reduced perceived exertion, and minimal exacerbation of LC-related symptoms.

In terms of occupational performance, participants with LC demonstrated significantly lower self-perceived satisfaction (3.10 ± 1.38) and performance (3.44 ± 1.44) compared to controls. These findings results align with previous findings using the COPM as an assessment tool (20). As underlined by Walker et al. (62), fatigue emerges as the symptom that is most markedly linked to functional impairment, which means that it adversely affects occupational performance. Moreover, this study observed a significant decline in the physical quality of life in LC patients (25.65 ± 7.98), consistent with O’Kelly et al. (63), while no significant differences were observed in mental quality of life. This divergence contrasts with Seeble et al. (64), who reported a decrease in both dimensions. Future studies should explore these differences in greater depth to delineate the multidimensional impact of LC.

An unexpected finding was the lack of correlation between heart rate and oxygen saturation with perceived exertion during ADLs in LC patients. This contrasts with Voorn et al. (66), who identified significant correlations between heart rate and perceived exertion in populations with neuromuscular diseases. However, the discrepancy may arise from differences in methodology, the authors mentioned above employed an aerobic training protocol with substantially higher physical activity levels compared to the ADLs assessed in this study. Moreover, the moderate-intensity nature of the tasks in this study may not have elicited sufficient cardiorespiratory responses to establish a measurable relationship. Future research should explore this observation by including higher-intensity activities or longitudinal designs to better capture dynamic changes in physiological responses during ADLs.

The present study also highlights that perceived exertion negatively and significantly correlates with physical quality of life and occupational performance in LC patients. These findings align with those of Calvo-Paniagua et al. (65), and underline the importance of Occupational Therapy interventions aimed at reducing the perceived exertion of the patient with LC when performing ADLs, as this will consequently lead to an increase in their physical quality of life and improved ADLs performance.

This study has several limitations that should be considered. First, the limited sample size and its restriction to participants from a single geographic region may constrain the generalizability and broader applicability of the findings. Furthermore, the potential influence of daily variability in symptoms and energy levels among individuals with LC was not accounted for in the study design. Symptom severity and perceived exertion are known to fluctuate throughout the day, which may have influenced participants’ performance and responses during assessments. Future research should address these limitations by including larger and more diverse samples across different territories to enhance the external validity of the results. Additionally, longitudinal studies are necessary to better understand the progression of the study variables over time and their potential interactions in individuals with LC. Incorporating assessments at different times of the day could also provide valuable insights into the daily variability of symptoms, contributing to a more comprehensive understanding of the impact of LC on daily life.

## Conclusion

Individuals with LC exhibit no significant differences in cardiorespiratory parameters during the performance of ADLs compared to those without LC. However, individuals with LC often report higher perceived exertion during ADLs, which may stem from chronic fatigue and post-exertional discomfort associated with the condition. This heightened perception of effort can contribute to a diminished physical quality of life among individuals with LC, highlighting the importance of addressing this aspect in their care. The novelty of these findings lies in the identification of a significant difference in perceived exertion despite similar cardiorespiratory parameters compared to individuals without LC, providing new insights into the challenges faced by this population.

Moreover, individuals with LC tend to demonstrate lower levels of satisfaction and performance in activities that involve a greater perception of exertion. Therefore, it becomes crucial for occupational therapy interventions to focus on reducing the perception of exertion during ADLs for patients with LC. By doing so, it is anticipated that their physical quality of life will improve, leading to enhanced performance in ADLs. This underscores the significance of tailored interventions aimed at mitigating the challenges posed by LC on daily functioning and overall well-being. Future research should further explore the multidimensional impact of LC, longitudinal studies and randomized controlled trials focusing on interventions to manage exertion perception and improve performance in ADLs will be valuable in refining occupational therapy strategies for this population.

## Data Availability

The original contributions presented in the study are included in the article/Supplementary material, further inquiries can be directed to the corresponding author.
