# Supplementary material for: Use of wearables to measure the effects of long COVID on activities of daily living and their relationship to perceived exertion, occupational performance, and quality of life
Source: Front Public Health. 2025 Feb 19;13:1519204. doi: 10.3389/fpubh.2025.1519204 (PMC11880940; doi:10.3389/fpubh.2025.1519204)
Supplement: Supplementary file 1 [file Data_Sheet_1.docx]

Supplementary Material

# ADLs ADMINISTRATION PROTOCOL

Initially, the day and time of the appointment will be arranged by email or phone with each participant. Every effort will be made to coordinate the scheduled times for participants. Before the appointment, the informed consent must be signed.

On the day of the appointment, the following steps will be followed:

- Collecting sociodemographic data (age, sex, height, and weight).
- Performing the four activities of daily living in the following order: setting the table, sweeping, putting on shoes, and climbing stairs. Participants may take their time and take all necessary breaks during the activity to complete it. A minimum break of 2 minutes will be taken between activities, taking advantage of the waiting time to measure the final heart rate (HR) and a maximum of 10 minutes according to the participant's needs. The following considerations will be followed:
  - Programming the accelerometer and placing it on the participant's hip.
  - One minute before starting each activity, with the patient sitting, the HR and % SpO2 will be measured with the pulse oximeter, in addition to administering the Borg scale. The HR and SpO2 will be collected using a pulse oximeter placed on the index finger.
  - Before starting the activities, participants will be instructed to perform them at the speed at which they normally do.
  - At the end of each activity, measure the HR, % SpO2, and administer the Borg scale again with the participant sitting.
  - After two minutes of rest with the participant sitting, measure the HR, % SpO2, and administer the Borg scale again after each activity.
  - Downloading accelerometer data after completing the four activities.
  - If the participant is unable to perform the four ADLs due to fatigue, it will be recorded as a consequence of the limitations caused by persistent COVID-19.
  - Not wearing the pulse oximeter during the activities.
- After completing the activities, the SF-12 and COPM questionnaires will be administered through an interview with the participant.

**MEASUREMENTS**

- Living room table: 140 cm (length) x 80 cm (width) x 70 cm (height).
- Utensils at a height of 60 cm.
- Plates and glasses at a height of 150 cm.
- Tablecloth and napkins at a height of 16 cm.
- Distance from the table to the countertop and kitchen furniture: 2 m.
- Total area to sweep is 3.25 m wide (from the apartment window) x 4.20 m long (from the apartment countertop).
- Trash bin 2 m away from the participant's seat, and the broom along with the dustpan 1 m away from the participant.

1. **SETTING THE TABLE FOR FOUR PEOPLE**

*What objects?*

- Table: 140 cm (length) x 80 cm (width) x 70 cm (height)
- Four chairs
- Tablecloth
- Four deep plates
- Four flat plates
- Four glasses
- Four spoons
- Four forks
- Four knives
- Four napkins

*How will they be positioned? Starting position?*

- The person initially sits at the table.
- The plates and glasses are inside the cabinet at approximately 1.50 m height.
- The utensils are inside the drawer at approximately 0.60 m height.
- The tablecloth and napkins are inside the drawer at approximately 0.16 m.
- The cabinet should be at the same distance from the table (approximately 2 m).

*Instructions*

You should start the activity seated in this chair at this table. You are expected to set the table for four people with a tablecloth, glasses, deep and flat plates, and utensils, including 4 spoons, 4 forks, 4 knives, and 4 napkins. You will find the glasses and plates in the cabinet, the utensils in the top drawer, and the tablecloth and napkins in the bottom drawer. The pitcher is in the cabinet. You should place the glasses and plates before the utensils. To finish the activity, you should leave the objects placed on the table and sit in the same chair as at the beginning of the activity. Have you understood?

*Observations*


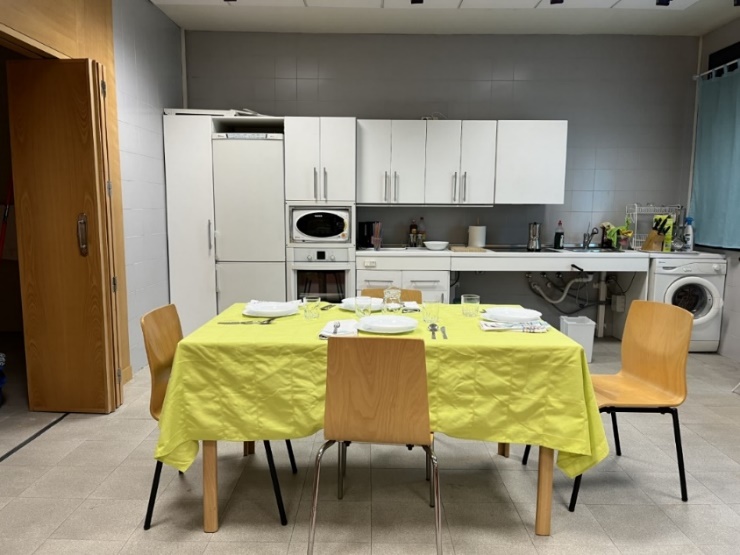

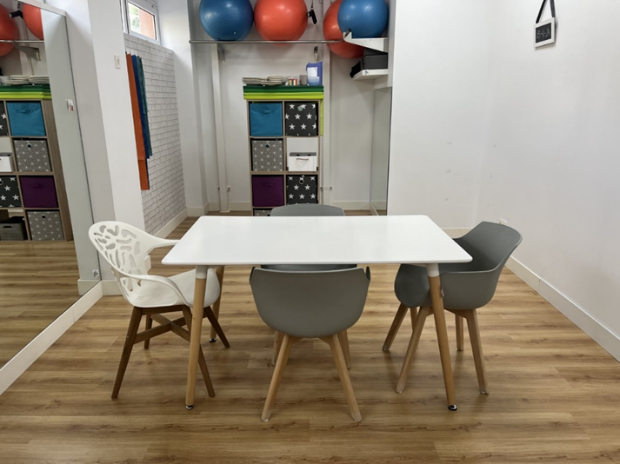
At the end of the activity, the subject must be in the starting position, and the glasses, plates, utensils, and napkins must be placed on the table. The plates, utensils, and glasses will be among other objects, but the same objects must always be present.

1. **SWEEPING**

*What objects?*

- Broom
- Dustpan with a long handle
- Open trash bin
- Table: 140 cm (length) x 80 cm (width) x 70 cm (height) and four chairs.

*How will they be positioned? Starting position?*

- The person initially sits on a chair at the table.
- The broom and dustpan are leaning against a wall 1 meter away from the person.
- The trash bin is in a corner of the room 2 meters away from the table.

*Instructions*

You should start the activity seated in this chair. You are expected to stand up, pick up the dustpan and broom leaning against the wall, sweep the kitchen floor, and throw the trash into the trash bin in the room. To sweep properly, you must move the chairs and place them back in their original positions. When you finish, you should leave the dustpan and broom in the same place and sit back in the chair exactly as it was at the beginning. Have you understood?

*Observations*


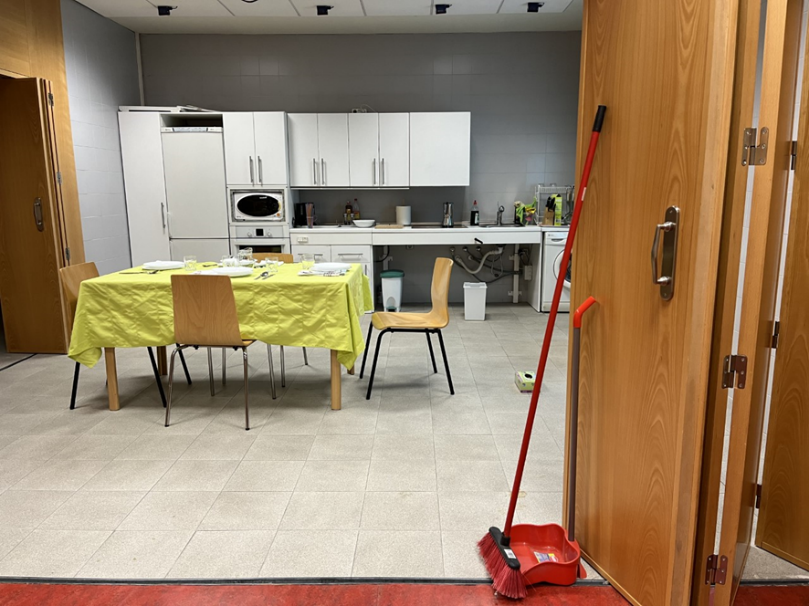
At the end of the activity, the subject must be in the starting position, as well as the objects used.


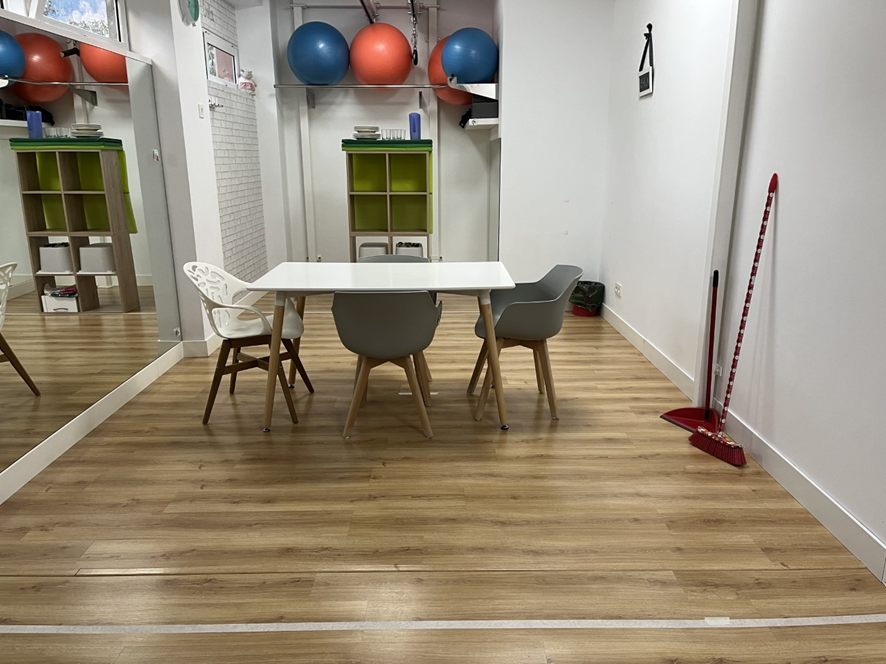


1. **PUTTING ON SOCKS AND SHOES**

*What objects?*

- Socks
- Shoes/sneakers with laces
- Chair

*How will they be positioned? Starting position?*

- The person initially sits on the chair in front of the objects and is barefoot. The chair is 2 meters away from where the shoes are located.
- The socks are next to the shoes, and the shoes are on the floor 2 meters away in front of the person.

*Instructions*

You should start the activity seated in this chair, barefoot. You must pick up the socks and shoes that are two meters away from the chair. You should then sit back down and put on the socks first and then the shoes. When you finish putting on the socks and shoes, you should remain seated without moving in the chair. Have you understood?

*Observations*


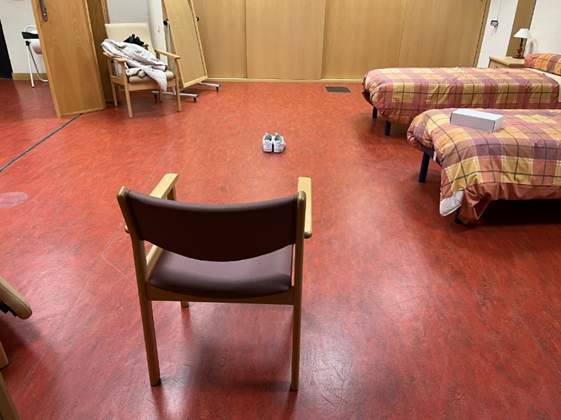
At the end, you should not make any further movements, simply remain seated in the chair. You should put on the socks first and then the shoes.


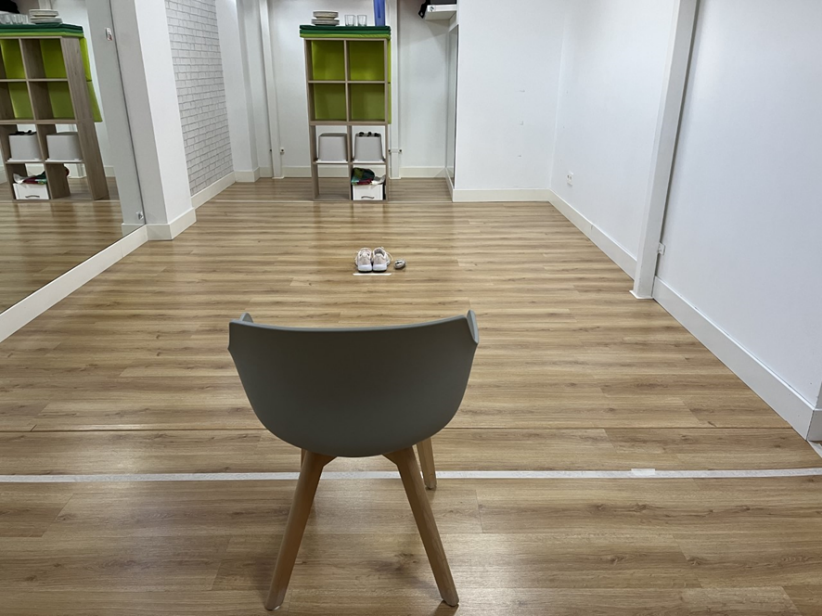


1. **CLIMBING STAIRS**

*What objects?*

- Stairs
- Marks on the floor at 1meter intervals

*How will they be positioned? Starting position?*

- The person initially stands upright 1 meter away from the beginning of the stairs (where the initial mark is located).
- The final mark is placed 1 meter away from the end of the stairs.

*Instructions*

You should start the activity 1 meter away from the stairs. You must move to the beginning of the stairs and climb all the steps. You can take all the stops and breaks you need until you climb all the steps. Once you have finished all the steps, you should move to the other mark placed 1 meter away from the stairs. Once at the mark, remain still. Have you understood?


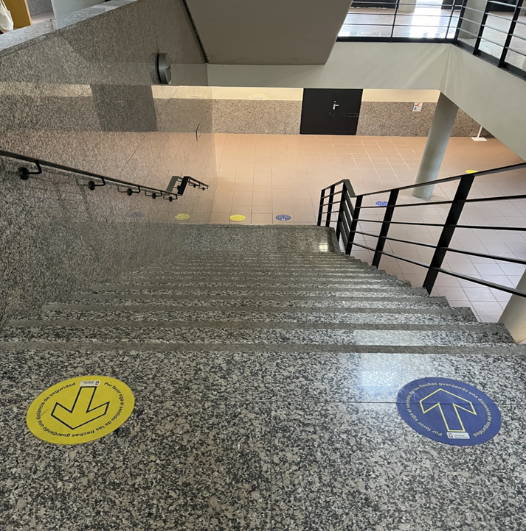
*Observations*

At the end, you should not make any further movements, simply stand still at the final mark.

# Supplementary tables

**Supplementary Table 1.** Comparison of means of study variables between people with and without long COVID (n=20).

|  | | | **CG**  **(n=10)** | **EG**  **(n=10)** | ***p* value** | **Effect size** |
| --- | --- | --- | --- | --- | --- | --- |
| **ADL_** **Table** | | |  |  |  |  |
|  | METs | | 1.44±0.55  [1.048-1.833] | 1.35±0.41  [1.056-1.647] | .650 |  |
|  | Total Time (sec) | | 108.90±17.53  [96.36-121.44] | 149.60±47.42  [115.68-183.52] | .004* | r (.49) |
|  | Light physical activity time (sec) | | 86.00±17.76  [73.29-98.71] | 125±39.51  [96.74-153.26] | .011* | r (.54) |
|  | Heart rate | |  |  |  |  |
|  |  | *At rest* | 69.20±8.92  [62.82-75.58] | 75.40±7.83  [69.80-81.00] | .116 |  |
|  |  | *Immediately after* | 77.30±8.73  [71.05-83.55] | 82.70±9.57  [75.85-89.55] | .204 |  |
|  |  | *After 2 minutes* | 72.50±11.93  [63.97-81.03] | 74.50±8.88  [68.14-80.86] | .676 |  |
|  | Oxygen saturation | |  |  |  |  |
|  |  | *At rest* | 97.10±2.28  [95.47-98.73] | 95.40±2.91  [93.32-97.48] | .116 |  |
|  |  | *Immediately after* | 95.80±4.16  [92.83-98.77] | 97.90±1.10  [97.11-98.69] | .165 |  |
|  |  | *After 2 minutes* | 94.60±5.25  [90.84-98.36] | 97.60±1.17  [96.76-98.44] | .106 |  |
|  | Borg Scale | |  |  |  |  |
|  |  | *At rest* | 0.00±0.00  [0.00-0.00] | 2.30±2.21  [0.72-3.88] | .002* | r (.59) |
|  |  | *Immediately after* | 0.80±1.23  [-0.08-1.68] | 4.00±1.83  [2.69-5.31] | .001* | r (.72) |
|  |  | *After 2 minutes* | 0.00±0.00  [0.00-0.00] | 3.30±1.89  [1.95-4.65] | <.001* | r (.35) |
| **ADL_** **Sweeping** | | |  |  |  |  |
|  | METs | | 1.70±0.80  [1.126-2.273] | 1.28±0.38  [1.009-1.549] | .473 |  |
|  | Total Time (sec) | | 154.10±25.70  [135.72-172.48] | 206.30±54.60  [167.24-245.36] | .014* | d (1.22) |
|  | Time light physical activity (sec) | | 108.00±42.63  [77.50-138.50] | 165±61.69  [120.87-209.13] | .027* | d (1.07) |
|  | Heart rate | |  |  |  |  |
|  |  | *At rest* | 72.30±8.50  [66.22-78.38] | 73.00±7.21  [67.84-78.16] | .909 |  |
|  |  | *Immediately after* | 81.00±7.42  [75.69-86.31] | 88.30±12.33  [79.48-97.12] | .126 |  |
|  |  | *After 2 minutes* | 66.50±23.88  [49.42-83.58] | 76.50±8.11  [70.70-82.30] | .161 |  |
|  | Oxygen saturation | |  |  |  |  |
|  |  | *At rest* | 96.40±2.41  [94.67-98.13] | 96.70±1.57  [95.58-97.82] | .745 |  |
|  |  | *Immediately after* | 96.50±3.66  [93.88-99.12] | 96.60±2.95  [94.49-98.71] | .847 |  |
|  |  | *After 2 minutes* | 96.00±3.16  [93.74-98.26] | 96.40±1.95  [95.00-97.80] | .939 |  |
|  | Borg Scale | |  |  |  |  |
|  |  | *At rest* | 0.00±0.00  [0.00-0.00] | 2.90±2.02  [1.45-4.35] | <.001* | r (.71) |
|  |  | *Immediately after* | 1.50±1.27  [0.59-2.41] | 5.60±1.90  [4.24-6.96] | .001* | r (.78) |
|  |  | *After 2 minutes* | 0.00±0.00  [0.00-0.00] | 3.80±2.49  [2.02-5.58] | .001* | r (.73) |
| **ADL_** **Shoes** | | |  |  |  |  |
|  | METs | | 1.95±0.78  [1.391-2.506] | 1.93±0.58  [1.510-2.344] | .450 |  |
|  | Total Time (sec) | | 57.60±22.41  [41.57-73.63] | 51.80±18.79  [38.36-65.24] | .910 |  |
|  | Time light physical activity (sec) | | 29.00±24.24  [11.66-46.34] | 28.00±16.86  [15.94-40.06] | .844 |  |
|  | Heart rate | |  |  |  |  |
|  |  | *At rest* | 72.20±10.56  [64.65-79.75] | 76.80±9.64  [69.91-83.69] | .322 |  |
|  |  | *Immediately after* | 78.70±9.24  [72.09-85.31] | 85.30±8.42  [79.28-91.32] | .112 |  |
|  |  | *After 2 minutes* | 71.10±11.69  [62.73-79.47] | 75.20±7.05  [70.16-80.24] | .355 |  |
|  | Oxygen saturation | |  |  |  |  |
|  |  | *At rest* | 96.80±2.89  [94.73-98.87] | 96.60±2.91  [94.52-98.68] | .695 |  |
|  |  | *Immediately after* | 96.20±2.35  [94.52-97.88] | 96.20±2.15  [94.66-97.74] | .969 |  |
|  |  | *After 2 minutes* | 97.60±1.83  [96.29-98.91] | 96.20±2.34  [94.52-97.88] | .159 |  |
|  | Borg Scale | |  |  |  |  |
|  |  | *At rest* | 0.00±0.00  [0.00-0.00] | 3.60±2.12  [2.08-5.12] | <.001* | r (.77) |
|  |  | *Immediately after* | 1.00±1.41  [-0.01-2.01] | 4.80±2.35  [3.12-6.48] | .002* | r (.70) |
|  |  | *After 2 minutes* | 0.00±0.00  [0.00-0.00] | 3.80±2.53  [1.99-5.61] | .001* | r (.73) |
| **ADL_** **Stairs** | | |  |  |  |  |
|  | METs | | 1.65±0.36  [1.390-1.911] | 1.83±0.48  [1.489-2.174] | .354 |  |
|  | Total Time (sec) | | 15.60±2.55  [13.78-17.42] | 19.40±4.25  [16.36-22.44] | .026* | d (1.08) |
|  | Time light physical activity (sec) | | 13.00±13.37  [3.43-22.57] | 14.00±13.50  [4.34-23.66] | .844 |  |
|  | Heart rate | |  |  |  |  |
|  |  | *At rest* | 72.70±10.47  [65.21-80.19] | 81.60±9.19  [75.02-88.18] | .058 |  |
|  |  | *Immediately after* | 97.10±9.46  [90.33-103.87] | 100.30±10.26  [92.96-107.64] | .494 |  |
|  |  | *After 2 minutes* | 72.90±9.34  [66.22-79.58] | 79.40±9.41  [72.67-86.13] | .138 |  |
|  | Oxygen saturation | |  |  |  |  |
|  |  | *At rest* | 96.60±1.84  [95.29-97.91] | 96.20±2.35  [94.52-97.88] | .877 |  |
|  |  | *Immediately after* | 98.70±4.11  [95.76-101.64] | 94.90±4.72  [91.52-98.28] | .137 |  |
|  |  | *After 2 minutes* | 97.00±1.94  [95.61-98.39] | 95.80±2.53  [93.99-97.61] | .260 |  |
|  | Borg Scale | |  |  |  |  |
|  |  | *At rest* | 0.00±0.00  [0.00-0.00] | 3.50±2.80  [1.50-5.50] | .002* | r (.66) |
|  |  | *Immediately after* | 1.90±1.45  [0.86-2.94] | 6.00±2.26  [438-7.62] | <.001* | d (2,16) |
|  |  | *After 2 minutes* | 0.00±0.00  [0.00-0.00] | 5.00±2.54  [3.18-6.82] | <.001* | r (.81) |
| **COPM** | | |  |  |  |  |
|  | Performance | | 9.27±0.44  [8.954-9.586] | 3.44±1.44  [2.411-4.469] | <.001* | r (.94) |
|  | Satisfaction | | 9.53±0.42  [9.226-9.834] | 3.10±1.38  [2.113-4.087] | <.001* | r (.95) |
| **SF-12** | | |  |  |  |  |
|  | Physical | | 54.99±3.44  [52.533-57.452] | 25.65±7.98  [19.941-31.363] | <.001* | r (.92) |
|  | Mental | | 50.68±10.89  [42.891-58.469] | 42.82±8.24  [36.927-48.722] | .086 |  |

*Data expressed in M±SD and [CI]; *p<0.05; CG=Control group; EG=Experimental group; d= Cohen's d; r= Rosenthal's r; ADL_ Table ="Setting the table"; ADL_ Sweeping= "Sweeping"; ADL_ Shoes = "Put on your shoes"; ADL_Stairs="Climb stairs"; COPM= Canadian Occupational Performance Measure; SF-12= Health Survey; METs=metabolic rate measurement unit.*

# Supplementary Table 2. Correlation between perceived exertion and heart rate in four activities of daily living in EG.

|  | **Heart rate** | | |
| --- | --- | --- | --- |
|  | *At rest* | *Immediately after* | *After 2 minutes* |
| **ADL_** **Table**  **Borg Scale** |  |  |  |
| *At rest* | -.151 |  |  |
| *Immediately after* |  | .306 |  |
| *After 2 minutes* |  |  | .509 |
|  |  |  |  |
| **ADL_** **Sweeping**  **Borg Scale** | **Heart rate** | | |
|  | *At rest* | *Immediately after* | *After 2 minutes* |
| *At rest* | .418 |  |  |
| *Immediately after* |  | .616 |  |
| *After 2 minutes* |  |  | .638*  (.047) |
|  |  |  |  |
| **ADL_** **Shoes**  **Borg Scale** | **Heart rate** | | |
|  | *At rest* | *Immediately after* | *After 2 minutes* |
| *At rest* | .353 |  |  |
| *Immediately after* |  | -.221 |  |
| *After 2 minutes* |  |  | .265 |
|  |  |  |  |
| **ADL_** **Stairs**  **Borg Scale** | **Heart rate** | | |
|  | *At rest* | *Immediately after* | *After 2 minutes* |
| *At rest* | .043 |  |  |
| *Immediately after* |  | .328 |  |
| *After 2 minutes* |  |  | .006 |

**p<0.05; ADL_ Table="Setting the table"; ADL_ Sweeping= "Sweeping"; ADL_ Shoes = "Put on your shoes"; ADL_Stairs="Climb stairs"*

**Supplementary Table 3. Correlation between perceived exertion and oxygen saturation in four activities of daily living in EG.**

| **ADL_** **Table** | **Oxygen saturation** | | |
| --- | --- | --- | --- |
|  | *At rest* | *Immediately after* | *After 2 minutes* |
| **Borg Scale** |  |  |  |
| *At rest* | -.323 |  |  |
| *Immediately after* |  | -.252 |  |
| *After 2 minutes* |  |  | .252 |
|  |  |  |  |
| **ADL_** **Sweeping** | **Oxygen saturation** | | |
|  | *At rest* | *Immediately after* | *After 2 minutes* |
| **Borg Scale** |  |  |  |
| *At rest* | -.709*  (.022) |  |  |
| *Immediately after* |  | -.471 |  |
| *After 2 minutes* |  |  | .087 |
|  |  |  |  |
| **ADL_** **Shoes** | **Oxygen saturation** | | |
|  | *At rest* | *Immediately after* | *After 2 minutes* |
| **Borg Scale** |  |  |  |
| *At rest* | -.416 |  |  |
| *Immediately after* |  | -.252 |  |
| *After 2 minutes* |  |  | -.868*  (.001) |
| **ADL_** **Stairs** | **Oxygen saturation** | | |
|  | *At rest* | *Immediately after* | *After 2 minutes* |
| **Borg Scale** |  |  |  |
| *At rest* | -.440 |  |  |
| *Immediately after* |  | -.554 |  |
| *After 2 minutes* |  |  | -.452 |

**p<0.05; ADL_ Table="Setting the table"; ADL_ Sweeping= "Sweeping"; ADL_ Shoes = "Put on your shoes"; ADL_Stairs="Climb stairs"*
